# The generalist tick *Ixodes ricinus* and the specialist tick *Ixodes trianguliceps* on shrews and rodents in a northern forest ecosystem– a role of body size even among small hosts

**DOI:** 10.1186/s13071-015-1258-7

**Published:** 2015-12-16

**Authors:** Atle Mysterud, Ragna Byrkjeland, Lars Qviller, Hildegunn Viljugrein

**Affiliations:** Department of Biosciences, Centre for Ecological and Evolutionary Synthesis (CEES), University of Oslo, P.O. Box 1066 Blindern, NO-0316 Oslo, Norway; Norwegian Veterinary Institute, P.O. Box 750 Sentrum, NO-0106 Oslo, Norway

**Keywords:** *Ixodes ricinus*, *Ixodes trianguliceps*, Ticks, Tick-borne diseases, Rodents, Voles, Shrews, Tick load, Host body size, Host selection, Norway

## Abstract

**Background:**

Understanding aggregation of ticks on hosts and attachment of life stages to different host species, are central components for understanding tick-borne disease epidemiology. The generalist tick, *Ixodes ricinus,* is a well-known vector of Lyme borrelioses, while the specialist tick, *Ixodes trianguliceps*, feeding only on small mammals, may play a role in maintaining infection levels in hosts. In a northern forest in Norway, we aimed to quantify the role of different small mammal species in feeding ticks, to determine the extent to which body mass, even among small mammals, plays a role for tick load, and to determine the seasonal pattern of the two tick species.

**Methods:**

Small mammals were captured along transects in two nearby areas along the west coast of Norway. All life stages of ticks were counted. Tick load, including both prevalence and intensity, was analysed with negative binomial models.

**Results:**

A total of 359 rodents and shrews were captured with a total of 1106 *I. ricinus* (60.0 %) and 737 *I. trianguliceps* (40.4 %), consisting of 98.2 % larvae and 1.8 % nymphs of *I. ricinus* and 91.2 % larvae, 8.7 % nymphs and 0.1 % adult females of *I. trianguliceps*. Due to high abundance, *Sorex araneus* fed most of the larvae of both tick species (*I. ricinus* 61.9 %, *I. trianguliceps* 64.9 %) with *Apodemus sylvaticus* (*I. ricinus* 20.4 %, *I. trianguliceps* 10.0 %) and *Myodes glareolus* (*I. ricinus* 10.9 %, *I. trianguliceps* 9.5 %) as the next most important hosts. Individual *A. sylvaticus* and *M. glareolus* had higher infestation intensity than *S. araneus*, while *Sorex minutus* had markedly lower infestation intensity. The load of *I. ricinus* larvae and nymphs was related to body mass mainly up to ~10 g, while the load of *I. trianguliceps* was less dependent of body mass. The load of *I. trianguliceps* was higher in spring than in fall, while the seasonal pattern was reversed for *I. ricinus* with higher loads in fall.

**Conclusions:**

Body mass was important for explaining load of *I. ricinus* mainly up to a body mass of ~10 g across a range of smaller mammalian hosts. Consistent with earlier work elsewhere in Europe, we found the highest tick infestation intensity on the wood mouse *A. sylvaticus*. However, this rodent species fed only 20.4 % of all *I. ricinus* larvae, while the much more abundant *S. araneus* fed 61.9 %. Our study emphasizes an important quantitative role of the common shrew *S. araneus* as a main host to *I. ricinus* larvae and to both *I. trianguliceps* larvae and nymphs. The partly seasonal distinct attachment pattern of *I. ricinus* and *I. trianguliceps* is evidence for niche separation.

## Background

The generalist sheep tick *Ixodes ricinus* in Europe is the principal vector of several pathogens causing disease in humans and livestock. This includes the pathogenic genospecies from the *Borrelia burgdorferi* sensu lato complex causing Lyme borrelioses [1] and the virus causing tick-borne encephalitis (TBE) [2] in humans, *Anaplasma phagocytophilum* causing tick-borne fever in livestock [3] and the protozoan *Babesia divergens* causing babesiosis in cattle. Most parasites [4], including *Ixodes* ticks [5–7], are typically aggregated on certain species and individuals, and an important element in understanding the epidemiology of the tick-borne diseases is identifying which species and individuals are feeding most of the ticks. The *I. ricinus* life cycle has three active life stages requiring a blood meal to moult into the next stage or to reproduce. The larvae and nymphs feed on a wide range of different sized hosts [8], while the adult female tick requires a blood meal from a large host to complete the life cycle. Small mammals are considered an especially important group due to their reservoir competence for pathogens (mainly *Borrelia afzelii*) causing Lyme borrelioses [9].

Ticks, vertebrates and associated pathogens form complex ecological networks [10]. Specialized tick species can also play a role in the epidemiology by maintaining high infection levels in the reservoir hosts, even if they do not act as vectors of disease to humans or livestock. One such example is the nest-dwelling rodent specialist, *Ixodes trianguliceps* that do not act as direct vectors for pathogens causing human or livestock diseases (as they reside in burrows) [11]. It has been shown that *I. trianguliceps* may play a role in maintaining high infection levels in the reservoir hosts with regards to *Babesia microti* [12] and *Anaplasma phagocytophilum* [13, 14]. Another tick species (*Ixodes neotomae*) specialized on rodents in California showed a similar transmission role with regard to *Borrelia* [15]. *I. trianguliceps* is associated with distinct *A. phagocytophilum* genotypes in central Europe [16] and Siberia [17]. All life stages of *I. trianguliceps* are expected to be on small mammal hosts, but in addition, body mass differences among these small mammals were also shown to have an effect on host selection of different stages of *I. trianguliceps* [18].

There are many studies of tick loads on small mammals from endemic areas of the USA [5, 19] and in central [20–22] and eastern [23–25] Europe, considered endemic areas for Lyme borrelioses. *I. ricinus* and Lyme borrelioses are currently spreading towards higher latitudes of Scandinavia [26–28]. Main hosts in Norway are known mainly from qualitative evidence [29]. There is one quantitative study, however it is limited to *Apodemus* spp. and ticks [30]. The objective of this study was twofold; (1) to quantify the load of *I. ricinus* and *I. trianguliceps* ticks on species of shrews and rodents in a northern area of Lyme borrelioses, and hence to understand the quantitative role of different species in feeding ticks, and (2) to determine the extent to which body mass could explain variation in tick load among species and individuals.

## Methods

### Study area

The study area is located in the western part of southern Norway, in Førde and Askvoll municipalities in Sogn & Fjordane county, close to the small town Førde (61°27’2˝ N, 5°51’15˝ E). The area lies mainly within the boreonemoral vegetation zone [31]. The bedrock is dominated by gneiss, granite, and other plutonic rock types, with limited coastal areas consisting of distinctive remnants of less modified sediments, such as conglomerate and sandstone. The region consists of mixed forests with deciduous woodland in the south facing slopes, with birch (*Betula* spp.), alder (*Alnus incana*), grass and herbs as the dominating vegetation. Other parts are dominated by Scots pine (*Pinus sylvestris*) together with planted Norway spruce (*Picea abies*). The study area is known for its mild winters and cold summers, with an average yearly precipitation of 2270 mm and an average temperature of 6.0 °C between 1961 and 1990 (http://met.no; Norwegian meteorological station no. 57170).

### Capture of small mammals

Rodents and shrews were captured along two transects in Angedalen (slightly inland; mean distance from fjord 9.1 km) and west of Førde city (termed Førde hereafter; coast; mean distance from fjord 2.8 km), Førde and Askvoll municipalities, Sogn & Fjordane county, Norway, during spring (2nd-5th of June) and fall (1st-4th of September) 2014. The trapping stations were spaced out with a minimum of 500 m in between to avoid depletion of the populations. Four traps were spaced out in the corners of a 15 m × 15 m square at each station according to the small quadrate method [32]. The traps were placed in natural structures or close to holes in the ground maximum 2 m from the square corners to enhance local capture probability. All traps were live traps of type “Ugglan” baited with carrots (for water) and oat (for food) on the first day of fieldwork. Food and water reserves would allow the rodents to survive for at least 24 h. The traps were baited the first day, and operated for three consecutive days. All traps were controlled every day. Small mammals captured were sacrificed (cervical dislocation) and transferred to an individual zip-lock plastic bag, marked with station number, trap number and date. All bags were stored in a freezer for later observation.

All small mammals were weighed and identified morphologically to species level. A representative subsample of animals was identified with assistance from a rodent specialist (Torbjørn H. Ergon). All ticks on the hosts were removed from the captured rodents and shrews, and identified morphologically to species level, and characterized by the life stages larva, nymph, adult female or adult male [33]. The identification of a representative subsample of ticks was checked by a tick specialist (Reidar Mehl [29]).

We define the term “prevalence” as the % of captured individuals with ticks, “intensity” as the number of ticks on a given host, and the “mean intensity” as the mean number of ticks among hosts with ticks [34]. We use the term parasite or tick “load” as a more general term for the whole pattern of parasitism including both prevalence and intensity.

### Questing tick collecting

Questing *I. ricinus* were sampled at every trapping station, once during spring and fall 2013—2014 using the cloth-lure technique [35]. A cotton towel (50*100 cm) was attached to a rod and dragged over the vegetation [36]. Each of the transects started from the middle of one side in the 15 m*15 m square, was 10 m long and 2 m wide, and was directed away from the square centre. A total area of 20 m^2^ was flagged and ticks were removed from the towel, counted and identified to life stages after every 2 m of flagging.

### Statistical analyses

Statistical analyses were conducted in R version 3.1.2 [37]. We used negative binomial models run in library glmmADMB [38]. We ran 4 separate models with load of *I. ricinus* and *I. trianguliceps* larvae and nymphs as response variables. Covariates were species, log-transformed body mass, location (Angedalen/Førde), and season (spring/fall), while trapping station was included as a random term. *Neomys fodiens* was excluded from analysis due to low sample size (Table 1). We used Akaike Information Criterion (AIC) for model selection using a combination of backward and forward selection procedures. Adding zero inflation did not improve model fit [39]. For questing tick data, we used abundance of nymphs as response, i.e. the number of ticks collected for each 20 m^2^ transect, and year (2013/1014), season (spring/fall) and location (Angedalen/Førde) as fixed effects with trapping station as a random term.

**Table 1:** An overview of samples sizes of small mammals captured during spring and fall 2014 in two study sites (Ang = Angedalen; For = Førde west) in Sogn & Fjordane county, Norway. The table shows abundances and percentages of *Ixodes ricinus* and *Ixodes trianguliceps* life stages on given small mammal species, and the prevalence (Prev) and mean intensity (Int) calculated across tick species and life stages

	Spring		Fall			*I. ricinus* larvae		*I. ricinus* nymphs		*I. trianguliceps* larvae		*I. trianguliceps* nymphs		*I. trianguliceps* adults		Prev	Int
	Ang	For	Ang	For	Sum	Sum	%	Sum	%	Sum	%	Sum	%	Sum	%		
*Apodemus flavicollis*		3		8	11	12	1.1			19	2.8	3	4.7	1	100	81.8	3.9
*Apodemus sylvaticus*	2	3	12	10	27	222	20.4	3	15	67	10.0	3	4.7			80.0	13.4
*Microtus agrestis*	1	1	11	7	20	47	4.3	12	60	43	6.4	2	3.1			82.6	5.8
*Myodes glareolus*	5	1	17	7	30	118	10.9	2	10	64	9.5	8	12.5			88.9	6.9
*Neomys fodiens*			2	1	3	6	0.6			8	1.2	1	1.6			66.7	7.5
*Sorex araneus*	2	4	123	103	232	672	61.9	3	15	436	64.9	47	73.4			71.0	7.0
*Sorex minutus*	2		6	23	31	9	0.8			35	5.2					34.2	4.9
Unknown			2	3	5												
Sum	12	12	173	162	359	1086		20		672		64		1			

## Results

We trapped 359 individuals of 7 species of small mammals (Table 1). They had in total 1106 *I. ricinus* (60.0 %) and 737 *I. trianguliceps* (40.4 %), consisting of 1086 larvae (98.2 %) and 20 nymphs (1.8 %) of *I. ricinus* and 672 larvae (91.2 %), 64 nymphs (8.7 %) and 1 adult female (0.1 %) of *I. trianguliceps*. The four species of rodents had high prevalence of ticks (>80 %), while *Sorex araneus* (71.0 %, host *n* = 232), *Neomys fodiens* (66.7 %, host *n* = 3) and particularly *Sorex minutus* (34.2 %, host *n* = 31) had lower prevalence. *S. araneus* fed most of the larvae of both tick species (*I. ricinus* 61.9 %, *I. trianguliceps* 64.9 %) with *Apodemus sylvaticus* (*I. ricinus* 20.4 % and *I. trianguliceps* 10.0 %) and *Myodes glareolus* (*I. ricinus*10.9 % and *I. trianguliceps* 9.5 %) as the next most important hosts (Table 2). In a simple model for load of *I. ricinus* larvae with species as categorical (controlling also for season), *A. sylvaticus* and *M. glareolus* had markedly higher load than *S. araneus*, while *S. minutus* had markedly lower load (Table 2).

**Table 2:** Parameter estimates of tick load in small mammals from negative binomial models. Baseline for species is *Sorex araneus*. Models for *I. ricinus* larvae were run excluding and (the best model) including body mass

	Estimate	Std. error	z	*p*
*I. ricinus* larvae				
*Excluding body mass*				
Intercept	0.4775	0.2314	2.06	0.039
*Apodemus flavicollis*	0.4029	0.6962	0.58	0.563
*Apodemus sylvaticus*	1.0826	0.3675	2.95	0.003
*Microtus agrestis*	−0.0725	0.3853	−0.19	0.851
*Myodes glareolus*	0.7528	0.3387	2.22	0.026
*Sorex minutus*	−2.8948	0.4927	−5.88	<0.001
Season (spring vs. fall)	−0.705	0.3618	−1.95	0.051
*Including body mass*				
Intercept	−2.6313	0.9461	−2.78	0.005
log (body mass)	1.5014	0.4446	3.38	0.001
*Apodemus flavicollis*	−1.4505	0.8787	−1.65	0.099
*Apodemus sylvaticus*	−0.0437	0.4891	−0.09	0.929
*Microtus agrestis*	−2.0303	0.6912	−2.94	0.003
*Myodes glareolus*	−0.7295	0.5382	−1.36	0.175
*Sorex minutus*	−1.4236	0.6455	−2.21	0.027
Season (spring vs. fall)	−0.9547	0.3593	−2.66	0.008
*I. ricinus* nymphs				
Intercept	−9.311	1.758	−5.30	<0.001
log (body mass)	2.36	0.616	3.83	<0.001
*I. trianguliceps* larvae				
Intercept	0.239	0.16	1.50	0.130
Season (spring vs. fall)	1.404	0.343	4.09	<0.001
*I. trianguliceps* nymphs				
Intercept	−7.227	1.959	−3.69	0.000
log (body mass)	1.705	0.876	1.95	0.052
*Apodemus flavicollis*	−3.841	1.385	−2.77	0.006
*Apodemus sylvaticus*	−1.140	1.090	−1.05	0.296
*Microtus agrestis*	−1.778	1.477	−1.20	0.229
*Myodes glareolus*	−0.647	1.207	−0.54	0.592
*Sorex minutus*	−20.20	26230.0	0.00	0.999
Location (Forde vs. Angedalen)	1.494	0.636	2.35	0.019

We found co-feeding *I. ricinus* larvae and nymphs on 8 individuals, all but one captured during fall. Only 3 of these individuals, *A. sylvaticus* (104 larvae, 2 nymphs), *M. agrestis* (20 larvae, 1 nymph) and *S. araneus* (21 larvae, 1 nymph), had more than 10 larvae together with at least one nymph (the conditions required for TBE persistence), while the remaining 5 individuals had 1–4 larvae. For *I. trianguliceps*, 21 individuals had co-feeding larvae and nymphs (4 in spring, 17 in fall), with only 2 individuals having more than 10 larvae together with at least 1 nymph (*M. glareolus*: 14 larvae, 2 nymphs; *S. araneus*: 15 larvae, 3 nymphs).

**Fig. 1:**
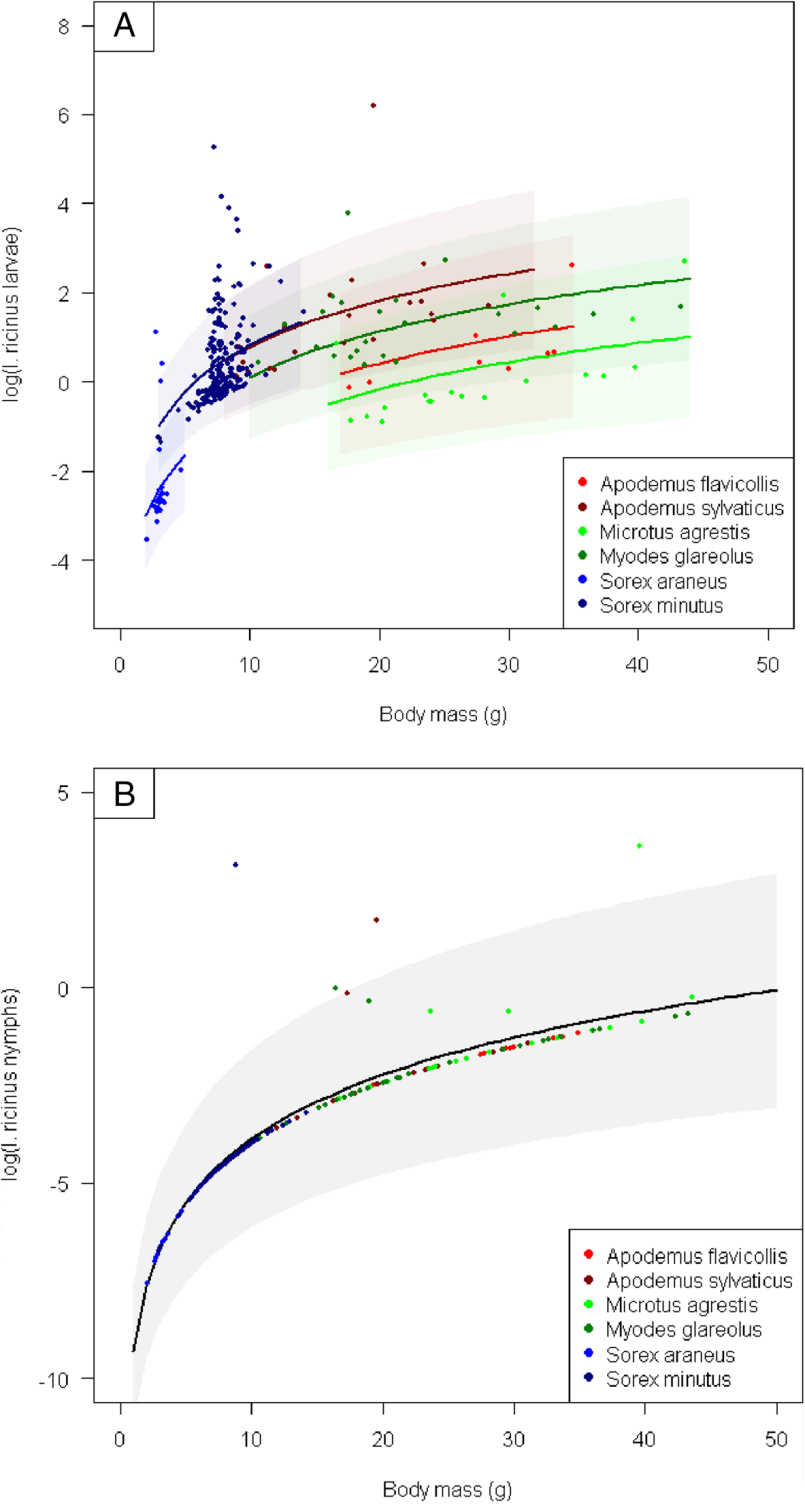
The number of *Ixodes ricinus* (**a**) larvae and (**b**) nymphs as a function of body mass in 6 species of small mammals captured along the west coast of Norway. Estimated effects are for season fall. Shaded areas are standard error

Model selection results are presented in Appendix 1: Table 3. The best model for load of *I. ricinus* larvae included (log) body mass, species and season, but did not include location. Load of *I. ricinus* larvae increased with (log) body mass, but note that the effect of body mass was mainly up to ~10 g, after that the relationship was virtually flat (Fig. 1a). There was some residual variation due to species (Fig. 1a), as *M. agrestis* and *S. minutus* had lower tick loads than expected for their body mass. Tick load was lower in spring than fall. The best model for load of *I. ricinus* nymph included only a positive effect of (log) body mass (Fig. 1b). Again, the effect was mainly due to an absence of *I. ricinus* nymphs on small mammals with a body mass below ~10 g. The best model for load of *I. trianguliceps* larvae included only season, with higher loads in spring compared to fall. If adding body mass, estimated effects were positive, but much weaker than for *I. ricinus* and not significant. The best model for load of *I. trianguliceps* nymphs included species, (log) body mass and location. *I. trianguliceps* nymph load tended to increase with body mass (*p* = 0.052), was lower in *Apodemus flavicollis* than expected from their size, and had higher loads in Førde compared to Angedalen. The effect of body mass was depending on inclusion of *S. minutus* (*n* = 31) without any nymphs.

**Fig. 2:**
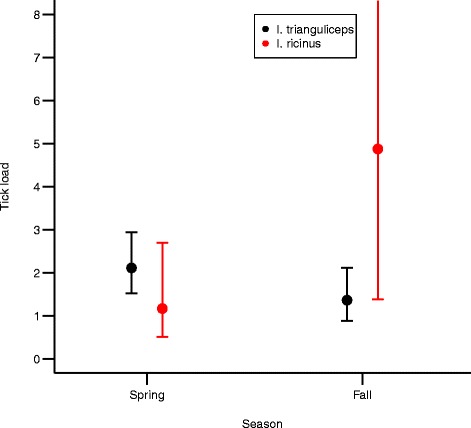
The number of larvae of *Ixodes ricinus* and *Ixodes trianguliceps* on small mammals in spring and fall along the west coast of Norway

We confirmed the reversed seasonal pattern fitting a model including larvae of both *I. ricinus* and *I. trianguliceps* in the same model. There was a highly significant interaction between season and tick species (z = 3.73, *p* < 0.001, Fig. 2)*.* There was a higher load of *I. ricinus* in fall compared to spring, while the reverse was found for *I. trianguliceps*.

The flagging data revealed far more *I. ricinus* nymphs in Førde (118 nymphs) than in Angedalen (4 nymphs, z = 4.14, *p* < 0.001), while there was no effect of season (z = 0.11, *p* = 0.91) or year (z = −1.53, *p* = 0.13).

## Discussion

Understanding the aggregation of different life stages of Ixodid ticks on different hosts has implications for tick population regulation and epidemiology. The level of co-feeding ticks is particularly important for TBE transmission, but also to some extent for other pathogens [7, 40, 41]. Further, both specialist and generalist Ixodid ticks can play a role in the epidemiology of tick-borne diseases [10]. Here, we document a different pattern of host selection by two tick species, one specialist and one generalist, on small mammals. We identify the most important small mammalian hosts for *I. ricinus* and *I. trianguliceps* ticks in a northern area with Lyme borrelioses, and we show that body mass is important for explaining tick load within species crossing a ~10 g body mass threshold, but less so across a range of mammalian hosts above this threshold mass. We also found differing seasonality in larval attachment of the two tick species.

The 4 species of rodents and 3 species of shrews found in this study are all known hosts of the generalist tick, *I. ricinus,* and the specialist tick, *I. trianguliceps* [18]. Several of them are known pathogen reservoirs in Europe [9], while so far only *A. flavicollis* and *A. sylvaticus* have been documented to carry *Borrelia afzelii* in Norway, likely due to few studies [30, 42]. For *I. ricinus*, *A. sylvaticus* was among the main tick hosts in Sweden [43], Poland [24, 25], Romania [23], Italy [7, 20], France [21] and Germany [22], and the species is highlighted as a link between woodland and field habitats [21, 24]. Also in our study, we found almost twice as high tick infestation intensity on *A. sylvaticus* (13.4 tick/host) compared to the other small mammal hosts, consistent with earlier work [44]. However, only 20.4 % of all *I. ricinus* larvae ticks were found on *A. sylvaticus*, while the common shrew *S. araneus* hosted 61.9 %. Shrews often dominate in abundance among small mammals, and *S. araneus* had the highest tick load estimate among host species when we controlled for body mass (Table 2). Their small size did, however, result in lower tick intensity per individual (7.5 tick/host) compared to *A. sylvaticus*, as earlier studies have shown for both *I. ricinus* [44] and *I. trianguliceps* [45]. Shrews have been highlighted as markedly underestimated as hosts to *I. ricinus* ticks in endemic areas of the UK [46] and for *I. scapularis* in USA [47], but have long been recognized as important hosts to *I. trianguliceps* [18]. Our study thus confirms an important role of shrews as a main host to *I. ricinus* larvae (Table 1). How such patterns may vary across years, due to differences in vegetation and humidity (affecting questing height), or phase of the population cycle of small mammals, remains to be established.

The simultaneous attachment of life stages on the same host is necessary for co-feeding transmission to occur [41]. Only 8 individuals (2.2 %) had co-feeding *I. ricinus* larva and nymph. Among these, one *A. sylvaticus*, one *M. agrestis* and one *S. araneus*, had more than 10 larvae and at least 1 nymph as required for TBE transmission. There are currently no reported case of TBE in this region of Norway, while TBEv is now found in the southernmost part of Norway [48]. Co-occurrence of life stages of *I. trianguliceps* was more common as expected for a rodent specialist. However, we found only a single adult *I. trianguliceps* (Table 1). The latter may at first sight seem surprising given that adults spend some 10 days feeding. Most likely this is due to low sample size, in particular for larger sized rodents.

Several mechanisms may give differences in tick load within and across species, and several of these mechanisms can in turn be linked to body size differences. The greater the size of the animal, the broader the front presented to the vegetation and so the greater the area it will sweep [8]. An animal covering the most ground will pick up the most questing ticks [18, 49], and in addition, home range size is well known to scale to body size when compared across large body size ranges [50, 51]. This may also depend on trophic niche, and shrews being insectivores may have a different relationship between home range size and body size [52]. Home range sizes of the rodents captured in our study are larger in *A. flavicollis* (0.75 ha [53]) and *A. sylvaticus* (1.3 ha [53]) than *M. glareolus* (~0.5 ha [54]), and even more so compared to *M. agrestis* (0.07 ha [53]), *S. araneus* (0.1 ha [55]), and *S. minutus* (0.05 ha [56], 0.2 ha [55]). *S. minutus* tend to have larger home ranges than *S. araneus* [18, 55], but nevertheless much fewer ticks. The pattern of tick load is hence not fully consistent with home range size across species. In chipmunks (*Tamias striatus*), tick load was linked to increased space use [57]. However, the relationship between home range size and body size is often less apparent at the intraspecific level (shown for large mammals [58, 59]), and space use may not explain the effect of body mass. Age and sex differences in size may be part of the intraspecific body size effect, and sex and age differences in immune defenses may play a role for tick load [60]. However, in *A. sylvaticus* and *M. glareolus*, the effect of sex on *I. ricinus* load was linked to body size rather than to sex [61, 62]. In addition to the direct effect of large body size on exposure, there might also be more active selection by ticks for different hosts. Larval deer ticks *I. scapularis* in the lab showed preference for white-footed mice (*Peromyscus leucopus*) over chipmunks [63]. Studies of attachment site selection of *I. ricinus* on larger hosts also suggest an active role of tick movements when on the host [64–66], and hence direct selection for larger individuals may be important.

Different species of ticks may be in competition on hosts [10]. In our study site, the two tick species were almost equally abundant (40 % *I. trianguliceps*, 60 % *I. ricinus*), but we found a different seasonality in their attachment pattern. We found higher load of *I. ricinus* in fall compared to spring, while the reverse was found for *I. trianguliceps* (Fig. 2). Such a seasonal disparate pattern of attachment between the two tick species, possibly to avoid competition by one or both of the species, may also lead to even more infection levels of pathogens over the season. Further studies are needed from areas with a less even composition of tick species (allopatry) in order to see if the seasonal patterns differ, and if such differences in temporal attachment pattern of different tick species might affect seasonal levels of infection in hosts.

### Ethics statement

Permissions to capture of rodents and shrews were given by the Norwegian Environment Agency (reference 2013/11201) and hence conform to the Norwegian laws and regulations.

## Conclusions

The common shrew *S. araneus* plays an important quantitative role as a main host to *I. ricinus* larvae and to both *I. trianguliceps* larvae and nymphs. Larger hosts had higher load of *I. ricinus*, but increased tick load with increased body mass appeared mainly up to a body mass of ~10 g. The attachment pattern of *I. ricinus* and *I. trianguliceps* was partly seasonally asynchronous. These results have implications for understanding tick-borne disease epidemiology in northern forests.

## Appendix 1

**Table 3:** Results of model selection of tick load. Best models used for inference are bolded. As there was only 1 *I. ricinus* nymph in spring, we avoided models with both season and location or species included

	Species	log (body mass)	Season	Location	df	AIC	ΔAIC
*I. ricinus* larvae	1	1	1		10	**1316.99**	**0.00**
	1	1	1	1	11	1317.98	0.99
	1	1			9	1321.60	4.61
	1	1		1	10	1322.58	5.59
	1		1		9	1326.47	9.48
	1				8	1328.09	11.10
	1			1	9	1328.90	11.91
		1	1		5	1337.54	20.55
		1	1	1	6	1339.03	22.04
		1			4	1342.21	25.22
		1		1	5	1343.74	26.75
			1		4	1367.10	50.11
				1	4	1368.55	51.56
			1	1	5	1368.74	51.75
*I. ricinus* nymphs		1			4	**107.16**	**0.00**
		1	1		5	108.09	0.93
		1		1	5	108.76	1.60
	1	1			9	113.41	6.25
			1		4	123.82	16.66
*I. trianguliceps* larvae			1		4	**1220.79**	**0.00**
			1	1	5	1220.98	0.19
		1	1	1	6	1221.15	0.36
		1	1		5	1221.16	0.37
	1	1	1	1	11	1225.09	4.31
	1	1	1		10	1225.39	4.60
	1		1		9	1225.62	4.84
		1			4	1231.99	11.20
	1	1		1	10	1234.22	13.44
	1	1			9	1234.37	13.58
				1	4	1237.86	17.07
	1				8	1240.43	19.65
*I. trianguliceps* nymphs	1	1		1	10	**271.60**	**0.00**
	1	1	1	1	11	273.56	1.96
		1		1	5	273.99	2.39
	1	1			9	275.03	3.43
	1	1	1	1	12	275.56	3.96
				1	4	276.74	5.13
		1			4	276.84	5.24
	1	1	1		10	277.00	5.39
			1	1	5	277.21	5.61
	1				8	277.51	5.91
	1		1		9	278.28	6.68
		1	1		5	278.79	7.19
